# Sitz bath with different concentrations of diluted povidone-iodine for prevention of perianal infection in patients with hematological malignancies undergoing chemotherapy: a randomized controlled trial in a tertiary hospital in China

**DOI:** 10.3389/fpubh.2026.1743662

**Published:** 2026-01-29

**Authors:** Yuqin Luo, Liyuan Long, Mei Yang, Li Zhou, Chen Chen, Jiaxing Li, Jinbo Fang, Yingli Wang

**Affiliations:** 1 Department of Hematology, West China Hospital, Sichuan University, Chengdu, Sichuan, China; 2 Department of Cardiology, West China Hospital, Sichuan University, Chengdu, Sichuan, China; 3 Cheng Du Shang Jin Nan Fu Hospital, West China Hospital, Sichuan University, Chengdu, Sichuan, China; 4 West China School of Public Health, Sichuan University, Chengdu, Sichuan, China; 5 West China Hospital, Sichuan University, Chengdu, Sichuan, China; 6 West China School of Nursing, Sichuan University, Chengdu, Sichuan, China

**Keywords:** chemotherapy, hematological malignancies, nursing, perianal infection, povidone-iodine, sitz bath

## Abstract

**Background:**

The incidence of perianal infection in patients with hematologic malignancies undergoing chemotherapy can reach 40–50%. Despite advances in medical technology and improved management strategies, the rate of perianal infection following chemotherapy remains substantial. Currently medicated sitz baths are not widely adopted in clinical practice due to their side effects and limited applicability. Povidone-iodine (PI) is a commonly used clinical broad-spectrum antiseptic, yet its efficacy in preventing perianal infection has not been adequately studied. Therefore, we aimed to evaluate the effect of different concentrations of diluted povidone-iodine in sitz baths on the incidence and severity of perianal infection, and to determine an appropriate concentration that minimizes adverse reactions.

**Methods:**

We recruited 180 patients from the Department of Hematology, West China Hospital, Sichuan University, between May 2021 and May 2023. Eligible patients received 14 days of perianal care consisting of either routine cleaning alone or routine cleaning plus sitz baths with povidone-iodine solution. They were randomly assigned to a control group (routine perianal cleaning and care, *n* = 60), a low-concentration intervention group (sitz bath with 1:200-diluted povidone-iodine, *n* = 59), and a high-concentration intervention group (sitz bath with 1:50-diluted povidone-iodine, *n* = 61).

**Results:**

A total of 171 patients aged 18–80 years undergoing chemotherapy for hematologic malignancies were included. The incidence of perianal infection significantly differed between the control group and both intervention groups with different concentrations (OR = 0.144, ARR = 0.221, 95% CI 0.038–0.054, *P* = 0.004; OR = 0.178, ARR = 0.197, 95% CI 0.053–0.590, *P* = 0.005). Compared with the control group (*n* = 60), the risk of perianal infection was reduced by 22.1% in the low-concentration group (*n* = 54) and by 19.7% in the high-concentration group (*n* = 57). No significant difference in infection incidence was observed between the two intervention groups (*P* > 0.05). The severity of perianal infection differed significantly between the intervention and control groups (*P* < 0.001). The risk of adverse reactions was 4.7 times higher in the high-concentration group than that in the low-concentration group (*P* = 0.001).

**Conclusions:**

Both 1:50- and 1:200-diluted povidone-iodine sitz baths are more effective than routine care in preventing perianal infections and can effectively reduce both the incidence and severity of perianal infections. However, the 1:50 dilution was associated with more perianal adverse events (AEs) compared with the 1:200 dilution.

**Trial Registration:**

It was registered in the Chinese Clinical Trial Registry (Registration number: ChiCTR 2000041073) on December 17, 2020.

## Introduction

Perianal infection is an acute suppurative infection that occurs in or around the soft tissue around the rectum and anal canal (1). Domestic and foreign studies have reported that the incidence of perianal infection in patients undergoing chemotherapy for malignant hematologic diseases can reach 40–50% (2). Despite the progress of medical technology and the improvement of management methods, the incidence is still as high as 27% and the recurrence rate is as high as 31% (3). The incidence of perianal infections in patients with hematological malignancies after chemotherapy remains high (4). Perianal infections prolongates the hospital stay of patients, increases the costs of treatment and delays the treatment of patients. In addition, the anal pain and discomfort caused by perianal infection will have a significant impact on the quality of life of patients, and in severe cases, sepsis may even occur and lead to death of patients (5). Currently, the methods for reducing perianal infection are mainly sitz baths, drug coating, and fumigation with traditional Chinese medicine (TCM), among which sitz baths have currently become a research hotspot in recent years (6).

Additionally, the methods mentioned above combined with potassium permanganate, traditional Chinese medicine (TCM), metronidazole etc. used in clinical administration have been used to reduce the incidence of perianal infection (7–9), but some problems have been found in the literature reported so far. For example, the compliance of patients using drug coating is often not high; The preparation method of TCM fumigation is complicated and difficult to implement in the ward. Moreover, the use of local antibiotic flushing, wet compress and other methods require cooperation, and must develop medical facilities, increase the cost of patient treatment, but also increase the workload of medical staff; In addition, the use of some new dressings increases the cost of materials and leads to low patient acceptance (7–9). In summary, the current methods used to control perianal infections have not been popularized due to side effects and limited scope of application. Povidone-iodine (PI) is a bactericidal agent having mild side effects and is a widely used broad-spectrum antibacterial agent (10), which is characterized by low irritation to skin tissue, strong bactericidal power, low side effects, wide application range, low price and convenient use, and has been favored by researchers. Current studies have confirmed the effectiveness of PI dilution sitz bath in the prevention and treatment of perianal infection during chemotherapy in patients with hematologic malignancies (10), but there are currently no randomized controlled clinical trials to verify the validity of the use of PI and its optimal concentration for the prevention and treatment of perianal infection in patients with hematologic malignancies.

The goals of this pragmatic randomized clinical study were to (1) explore the impact of a sitz bath with diluted PI on the incidence and severity of perianal infection in patients with hematological malignancies during chemotherapy and (2) compare perianal adverse reactions between groups receiving sitz baths with different concentrations of diluted PI.

## Methods

### Study design

This was a single-center, blinded outcome assessor-based, parallel, placebo-controlled, dose-finding, randomized, controlled, superiority clinical trial conducted at the Department of Hematology, West China Hospital, Sichuan University, China, from May 2021 to July 2023.

### Patients

***Inclusion eligible***

The patients were 18–80-year-old patients with hematologic malignancies undergoing chemotherapy who had high-risk factors for perianal infection but did not develop perianal infection and had an estimated hospital stay of ≥14 days. Patients with high-risk factors were those with a history of perianal infection and those with ≥3 of the following risk factors: age < 60 years, history of hemorrhoids, history of anal fissure, susceptibility to diarrhea, receipt of leukocyte promotion therapy before chemotherapy, and receipt of a high dose of chemotherapy (11).

#### Exclusion criteria

The patients with chronic leukemia, multiple myeloma, Crohn's disease, allergy to iodine or PI, late pregnancy, postpartum 2 weeks, vaginal bleeding, and acute pelvic infection. Additionally, patients with mental disorders, those who were unable to cooperate with treatment, those not completing the trial due to other reasons, and those with incomplete data were excluded.

#### Exemption criteria

The patients who were expected diarrhea, a white blood cell count ≥1 × 10^9^/L within 1 week of chemotherapy, and no diarrhea after chemotherapy.

#### Dropout criteria

The patients who were serious adverse reactions, lack of efficacy, need to change treatment plan, automatic withdrawal, loss of contact, or failure to complete the test for other reasons.

### Sample size calculation

This study adopted a randomized controlled design. The main evaluation index was the incidence of perianal infection, which was regarded as counting data. The sample size calculation method of the multi-group rate comparison was adopted, and the sample size was calculated using PASS 2022. For the sample size calculation, we used the chi-square test using contingency tables. The two-sided type *I* error probability (α) was set at 0.05, with a test power (1 – β) of 0.9; λ = 12.6, and 2 degree of freedom. The sample size allocation ratio among groups was set to 1:1:1. Based on preliminary data, the rate of perianal infection in patients with a history of perianal infection was 31.5%, and the lowest incidence observed in patients with three high-risk factors was 26.7%. In this study, the incidence of perianal infection in patients included in the standard care group after chemotherapy was expected to be 25%. The incidence of perianal infection can be reduced to < 5% using sitz baths containing PI (10). The incidence of perianal infection in the two intervention groups was expected to be 5% with a sample size of *n*_1_ = *n*_2_ = *n*_3_ = 50. Simultaneously, it was necessary to consider the low compliance of patients and loss to follow-up. The final sample size included in this study was 180, and when the sample size was sufficient, the trial was stopped.

The trial followed the Consolidated Standards of Reporting Trials (CONSORT) guidelines. The detailed methods and statistical analysis plan for the trial can be found in the previously published protocol (Supplementary 1) (11). The study was conducted in accordance with the Declaration of Helsinki. Written informed consent was obtained before randomization. This study was approved by the Ethics Committee on Biomedical Research at the West China Hospital of Sichuan University (approval code: NO. 2020-1170). It was also registered in the Chinese Clinical Trial Registry on December 17, 2020 (registration ID: ChiCTR 2000041073), and the trial registration data are shown in Supplementary 1.

Through discussions with research team members, we established a Study Steering Committee with an independent clinical chair and additional members. The following amendments to the original protocol were implemented following Study Steering and Ethics Committee approval: (1) An interim analysis in 113 recruits in August 2022 found that there was no statistical significance between the intervention groups; therefore, we hypothesized that increasing the difference in the concentration between the intervention groups would achieve the desired goal. Therefore, we reduced the four parallel groups to three and changed the concentration of the iodine sitz bath in the experimental group from 1:100, 1:200, and 1:300 to 1:50 and 1:200. (2) The sample size decreased from 268 to 180 participants because of a reduction in the trial group in August 2022. (3) In August 2022, an interim analysis revealed that the results of anal swab bacterial culture were not statistically significant. Furthermore, two rounds of expert consultation concluded that the assessment of pain lacked specificity as an endpoint for this study. Considering these findings alongside funding constraints, the decision was made to omit these two outcome indicators. The final retained primary outcomes were the incidence of perianal infection, the severity of perianal infection, and the incidence of perianal adverse events (AEs).

### Randomization, assignment, and blinding

Before study initiation, the members of the research group were trained for familiarization with the research content to ensure consistency in data collection. Researchers in the Department of Hematology conducted and coordinated the trial and were responsible for collecting all trial data. Members of an independent adjudication committee who were blinded to the study group assignments reviewed the research process. The accuracy of the collected data was monitored by investigators from the Data Management Safety Committee (DMSC).

A simple random grouping method was used to number the patients according to the sequence of entering the experiment, to generate a random number for each subject (using the random number table in the Appendix of Practical Health Statistics of Peking University) and to sort the random numbers from small to large. The top 33.3% were in Group A, followed by Groups B and C. The corresponding experimental and control groups were entered according to a random number corresponding to the number of patients. Group C was treated with traditional perianal cleaning care, and groups A and B were treated with 1:50- and 1:200-diluted PI sitz baths, respectively, on the basis of perianal cleaning care.

A statistical analyst generated the allocation sequence. The generated random numbers were placed in sequentially coded, opaque, sealed envelopes. When the subjects met the inclusion criteria, envelopes with the same number were opened according to the patient numbers, so that the subjects could be assigned to the corresponding group. Physicians and nurses assessing the perianal conditions and statisticians were unaware of the study group assignments. During the analysis phase, the three study groups were identified as groups A, B, and C, and the results were incorporated into a final report by the writing group before the data were unblinded.

### Intervention measures

Using a randomized network-based grouping, patients were allocated at a ratio of 1:1:1 and provided 14 days of regular cleaning or regular cleaning plus sitz baths with a PI solution with a concentration of 1:50 (40 ml of 5% PI mixed with 2,000 ml of warm water) or 1:200 PI solution (10 ml of 5% PI mixed with 2,000 ml of warm water).

#### Control group

The patients in the control group received 14 days of perianal cleaning care, including a perianal cleaning care package consisting of a basin, a water thermometer, a towel, a 2,000-ml measuring cup, and training on perianal cleaning. Starting from the day of chemotherapy, the perianal area was washed with warm water before getting up in the morning, before going to bed, and after defecation, to keep the perianal area clean and dry. Particularly, the cleaning method involved completely immersing the perianal skin in 2,000 ml warm water at 40–45 °C, gently wiping the perianal skin with a towel for 1–2 min, and drying the perianal skin. Water was provided by the inpatient department. Two trained observers recorded the perianal symptoms on the day before chemotherapy, on the 7th and 14th days of the study, and on the day before discharge. If a patient developed perianal symptoms outside the observation period, the investigator was required to promptly assess and record them in detail (Supplementary 2).

#### Intervention group

The patients were provided with the same perianal sitz bath care package as the control group and a 20 ml or 50 ml measuring cup, except that the warm water cleaning was changed to a sitz bath containing PI of different concentrations. The patient was trained in the perianal cleaning and perianal sitz bath methods. The 500 ml 5% PI solution was produced by Guangdong Kelun Pharmaceutical Co., Ltd. (National drug approval number H44023381). Patients were randomly assigned to sitz baths based on the accurate PI concentration. The duration of sitz bath was 14 days, and the frequency of the sitz bath was twice a day, once in the morning and once in the evening; both were performed after cleaning the anus before getting up in the morning and going to bed. In addition, patients with severe anemia or moderate-to-severe self-care ability needed to be accompanied by family members for the sitz bath. The temperature of the sitz bath in this group was similar to that of the control group, i.e., 40 °C−45 °C, and the sitz bath duration was 10 min. The height of the sitz bath was 40 cm, which was consistent with the height of the ward seat.

The concentrations of PI in the sitz bath were 1:50 (40 ml of 5% PI mixed with 2,000 ml of warm water) and 1:200 (10 ml of 5% PI mixed with 2,000 ml of warm water). After each sitz bath, the patient recorded the start time and temperature of the sitz bath on the sitz bath inspection form. Two specially trained personnel observed and recorded the perianal symptoms on the day before chemotherapy, on days 7th and 14th days of the study, and on the day before discharge. If the patient developed perianal symptoms outside the observation period, the researcher promptly evaluated and recorded the symptoms in detail (Supplementary 2).

### Study outcomes

The primary endpoint of this study was the incidence of perianal infection during hospitalization. Perianal infection was clinically defined as the presence of localized signs of inflammation (e.g., redness, swelling, heat, pain) or infection (e.g., purulent discharge, abscess) in the perianal region (12). This was assessed by two independent researchers through direct examination at four time points: on day 0 (baseline), day 7, day 14 of chemotherapy, and prior to discharge. A patient was considered to have reached the primary endpoint if a perianal infection was diagnosed at any of these assessments. Conversely, the absence of infection at all evaluations prior to discharge was recorded as no infection.

The secondary endpoints of the study were (1) the severity of perianal infection and (2) the incidence of adverse events (AEs).

- Infection Severity: If a perianal infection occurred, its severity was independently assessed and confirmed by two researchers according to a predefined four-grade scale (12):

Grade 0: Normal perianal skin; clean, dry anal mucosa without erythema or swelling.

Grade I: Localized erythema, swelling, warmth, and tenderness of the perianal skin.

Grade II: Marked redness, swelling, heat, and pain, with abscess formation and palpable fluctuance at the lesion site.

Grade III: Skin ulceration, necrosis, infected or bleeding wounds, or sinus tract formation.

- AEs: In this study, AEs primarily refer to adverse reactions following perianal medication administration. This includes any unintended response to the drug that is inconsistent with the treatment purpose and causes discomfort or harm to the patient, which was recorded irrespective of its perceived relationship to the study intervention. AEs were categorized as:

Procedure-related: Incidents associated with the sitz bath procedure (e.g., burns, slips/falls);

Local Symptoms: Patient-reported perianal symptoms such as burning, desquamation (peeling), dryness, hyperpigmentation, or urinary irritation.

AEs were captured through a combination of passive reporting (where patients spontaneously reported symptoms) and active assessment (where researchers specifically inquired during scheduled visits on days 0, 7, and 14).

### Statistical analysis

The validity analysis of the study will be based mainly on intention-to-treat (ITT; all randomized cases). All data were statistically analyzed using the R statistical software package (v4.0.2, R Core Team 2020). The statistical tests were two-sided, and statistical significance was set at *P* < 0.05. First, the Shapiro–Wilk test was used to test whether the quantitative data followed a normal distribution. The counting data are presented as proportions. The measurement data conforming to a normal or nearly normal distribution were presented as the mean ± standard deviation, and the counting data not conforming to a normal distribution were presented as the median and quartile spacing.

The main outcome measure of this study was the occurrence of perianal infections. Binary logistic regression was used to compare differences between the low- and high-concentration groups and the control group. Secondary outcome indicators were the degree of perianal infection and perianal adverse events. The severity of perianal infection was tested using the Kruskal–Wallis rank sum test, and perianal adverse events were described and analyzed using frequencies. The statistical results are based on a bilateral test, and α = 0.05 was the significance level.

## Results

Sitz baths containing PI could reduce the incidence of perianal infections in patients with hematologic malignancies undergoing chemotherapy.

A low concentration of diluents (sitz bath concentration of 1:200) caused fewer adverse reactions in patients. The specific results are described below.

### Participants of the study

**Figure 1:**
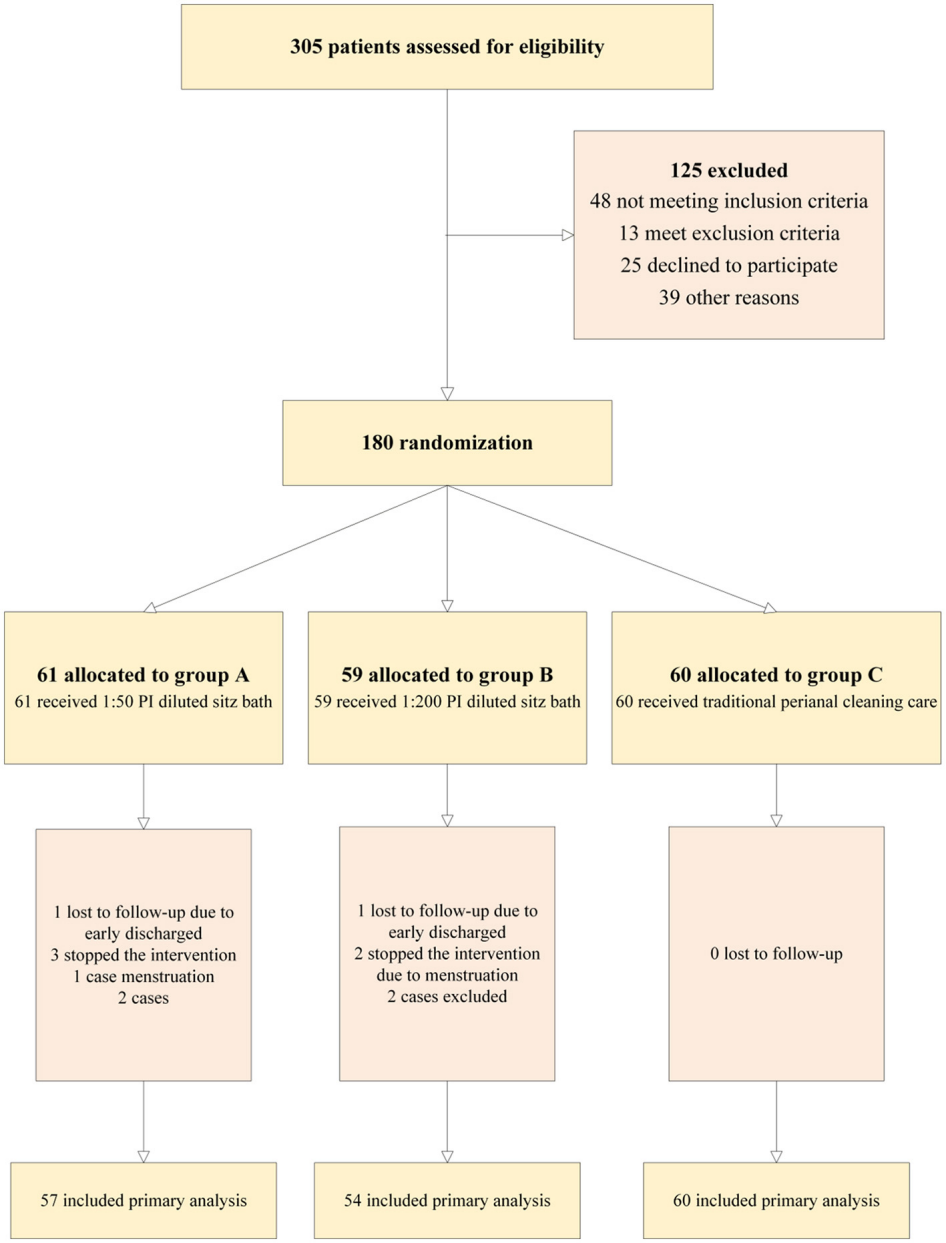
CONSORT diagram of the study.

A total of 180 subjects were enrolled, including 60 in the randomized control group; 59, low-concentration group; and, 61, high-concentration group. In the high-concentration sitz bath group, four patients dropped out due to menstruation, early discharge, or worsening condition. In the low-concentration sitz bath group, two patients were excluded due to a white blood cell count >1 × 10^9^/L 1 week after chemotherapy, and three patients dropped out due to menstrual period and early discharge. Therefore, a total of 171 patients completed the study: 60 in the control group, 54 in the low-concentration sitz bath group, and 57 in the high-concentration sitz bath group. The research flowchart is shown in Figure 1.

Of the 171 patients ultimately enrolled, there were 22 men and 38 women in the control group, with a mean age of 43.2 ± 13.9 years. In the low-concentration group, there were 15 men and 39 women, with an average age of 38.0 ± 15.9 years. In the high-concentration group, there were 20 men and 37 women, with an average age of 37.8 ± 12.6 years. Additionally, the difference in education levels among the three groups was statistically significant (*P* < 0.05). Other general information was balanced and comparable among the three groups (Table 1).

**Table 1:** Baseline characteristics.

**Characteristics**	**Control group (*n* = 60)**	**Low concentration intervention group (*n* = 54)**	**High concentration intervention group (*n* = 57)**	**Statistic**	***P-*value**
**Sex, no. (%)**					
Male	22 (36.7)	15 (27.8)	20 (35.1)	1.129	0.569
Female	38 (63.3)	39 (72.2)	37 (64.9)		
**Age, no. (%)**					
< 60	55 (91.7)	49 (90.7)	54 (94.7)	0.701^a^	0.767
≥60	5 (8.33)	5 (9.26)	3 (5.26)		
**Educational level, no. (%)**					
Junior high school and below	25 (41.7)	7 (13.0)	22 (38.6)	8.145^b^	0.017
High school	24 (40.0)	31 (57.4)	17 (29.8)		
University and above	11 (18.3)	16 (29.6)	18 (31.6)		
**Marital status, no. (%)**					
Married	42 (70.0)	30 (55.6)	35 (61.4)	5.339^a^	0.385
Unmarried	16 (26.7)	19 (35.2)	16 (28.1)		
Divorce	2 (3.33)	5 (9.26)	6 (10.5)		
**Primary caregiver, no. (%)**					
Spouse	30 (50.0)	17 (31.5)	33 (57.9)	9.843^a^	0.043
Parents	16 (26.7)	24 (44.4)	17 (29.8)		
Children, siblings	14 (23.3)	13 (24.1)	7 (12.3)		

^a^*x*^2^, ^b^*H*-value.

### Disease-related characteristics

Most patients in the three groups suffered acute myeloid leukemia (AML), accounting for 65.0%, 57.4%, and 73.7%, respectively. Over 70% of the patients in the three groups did not relapse and were refractory to treatment. In the control group, 36.7% of patients had diarrhea during chemotherapy, and 13.3% had diarrhea for more than 3 days. In the low-concentration group, 22.2% of the patients had diarrhea during treatment and 3.7% had diarrhea for more than 3 days. In the high-concentration group, 15.8% of the patients developed diarrhea and 1.8% had diarrhea for more than 3 days. The occurrence and duration of diarrhea among the three groups of patients (*P* < 0.05) significantly differed, and the outcome indicators were compared under the premise of controlling these two variables. The comparison of other disease characteristics among the three groups showed no significant differences (*P* > 0.05; Table 2).

**Table 2:** Disease related characteristics.

**Essential information**	**Control group (*n* = 60)**	**Low concentration intervention group (*n* = 54)**	**High concentration intervention group (*n* = 57)**	**Statistic**	***P*-value**
**Diagnose, no. (%)**					
AML^b^	39 (65.0)	31 (57.4)	42 (73.)	6.189^a^	0.185
ALL^c^	11 (18.3)	16 (29.6)	12 (21.1)		
Lymphoma	10 (16.7)	7 (13.0)	3 (5.26)		
**Disease stage, no. (%)**					
Induction chemotherapy	30 (50.0)	29 (53.7)	25 (43.9)	1.1037^a^	0.576
Consolidation chemotherapy	30 (50.0)	25 (46.3)	32 (56.1)		
**Recurrence or not, no. (%)**					
No	46 (76.7)	43 (79.6)	42 (73.7)	0.547^a^	0.761
Yes	14 (23.3)	11 (20.4)	15 (26.3)		
**ADL score, no. (%)**					
≤ 60	3 (5.0)	3 (5.6)	2 (3.5)	0.282^a^	0.907
>60	57 (95.0)	51 (94.4)	55 (96.5)		
**Diarrhea, no. (%)**					
No	38 (63.3)	42 (77.8)	48 (84.2)	7.127^a^	0.028
Yes	22 (36.7)	12 (22.2)	9 (15.8)		
**Duration of constipation (days), no. (%)**					
≤ 3	52 (86.7)	52 (96.3)	56 (98.2)	7.488^a^	0.039
>3	8 (13.3)	2 (3.7)	1 (1.8)		
**Constipation, no. (%)**					
No	37 (61.7)	35 (64.8)	37 (64.9)	0.172^a^	0.917
Yes	23 (38.3)	19 (35.2)	20 (35.1)		
**Constipation persists, no. (%)**					
≤ 3	51 (85.0)	50 (92.6)	50 (87.7)	1.615^a^	0.446
>3	9 (15.0)	4 (7.4)	7 (12.3)		
Diabetes, no. (%)
No	54 (90.0)	47 (87.0)	52 (91.2)	0.544^a^	0.762
Yes	6 (10.0)	7 (13.0)	5 (8.8)		
**Hemorrhoids, no. (%)**					
No	14 (23.3)	9 (16.7)	12 (21.1)	0.794^a^	0.672
Yes	46 (76.7)	45 (83.3)	45 (78.9)		
**Anal fissure, no. (%)**					
No	36 (60.0)	26 (49.1)	35 (61.4)	1.897^a^	0.335
Yes	24 (40.0)	27 (50.9)	22 (38.6)		
**Perianal infection, no. (%)**					
No	28 (46.7)	33 (61.1)	28 (49.1)	2.669^a^	0.263
Yes	32 (53.3)	21 (38.9)	29 (50.9)		

^a^χ^2^; ^b^acute myeloblastic leukemia; ^c^acute lymphoblastic leukemia.

### Comparison of laboratory test results

The laboratory test results did not significantly differ between the three groups before chemotherapy and on days 14 and 21 of chemotherapy (*P* > 0.05); however, there was a statistically significant difference in the laboratory test results between the three groups on the 7th day of chemotherapy (*P* < 0.05), which may be related to individual differences and other reasons (Table 3). In addition, the trend in blood cell changes before and after chemotherapy was the same in all three groups, and the duration of myelosuppression and recovery time did not differ significantly. Based on the above analysis, the three groups were comparable under the premise of adjust for the two variables of diarrhea and diarrhea duration.

**Table 3:** Laboratory test results.

**Inspecting item**	**Control group (*n* = 60)**	**Low concentration intervention group (*n* = 54 )**	**High concentration intervention group (*n* = 57 )**	**Statistic**	***P-*value**
**Albumin count before chemo (g/L), no. (%)**					
< 30	3 (5.0)	1 (1.9)	1 (1.7)	2.172^b^	0.338
30–35	8 (13.3)	10 (18.5)	5 (8.8)		
≥35	49 (81.7)	43 (79.6)	51 (89.5)		
Proportion of primitive cells before chemo, no. (%)	0.00 [0.00, 7.25]	0.00 [0.00, 17.0]	0.00 [0.00, 5.00]	0.684^b^	0.71
**White blood cell count before chemo (10** ^9^ **/L), no. (%)**					
< 1	8 (13.3)	2 (3.7)	5 (8.8)	4.463^b^	0.107
1–3.5	23 (38.3)	19 (35.2)	13 (22.8)		
≥3.5	29 (48.4)	33 (61.1)	39 (68.4)		
**WBC**^c^ **on the 7th day of chemo (10**^9^**/L), no. (%)**					
< 1	31 (51.7)	8 (14.8)	16 (28.1)	19.131^b^	< 0.001
1–3.5	18 (30.0)	21 (38.9)	17 (29.8)		
≥3.5	11 (18.3)	25 (46.3)	24 (42.1)		
**WBC on the 14th day of chemo (10** ^9^ **/L), no. (%)**					
< 1	38 (63.3)	22 (40.7)	37 (64.9)	5.727^b^	0.057
1–3.5	12 (20.0)	26 (48.1)	15 (26.3)		
≥3.5	10 (16.7)	6 (11.1)	5 (8.8)		
**WBC on the 21st day of chemo (10** ^9^ **/L), no. (%)**					
< 1	14 (23.3)	14 (25.9)	19 (33.3)	1.050^b^	0.592
1–3.5	30 (50.0)	22 (40.7)	22 (38.6)		
≥3.5	16 (26.7)	18 (33.4)	16 (28.1)		
**Neutrophil before chemo (10** ^9^ **/L), no. (%)**					
< 0.5	18 (30.0)	13 (24.1)	13 (22.8)	2.239^b^	0.327
0.5–1.5	14 (23.3)	9 (16.7)	10 (17.5)		
≥1.5	28 (46.7)	32 (59.3)	34 (59.6)		
**Neutrophil count on the 7th day of chemo (10** ^9^ **/L), no. (%)**					
< 0.5	29 (48.3)	13 (24.1)	21 (36.8)	6.656^b^	0.036
0.5–1.5	10 (16.7)	13 (24.1)	6 (10.5)		
≥1.5	21 (35.0)	28 (51.8)	30 (52.6)		
**Neutrophil count on the 14th day of chemo (10** ^9^ **/L), no. (%)**					
< 0.5	41 (68.3)	26 (48.1)	41 (71.9)	4.998^b^	0.082
0.5–1.5	6 (10.0)	17 (31.5)	6 (10.5)		
≥1.5	13 (21.7)	11 (20.4)	10 (17.5)		
**Neutrophil count on the 21st day of chemo (10** ^9^ **/L), no. (%)**					
< 0.5	19 (31.7)	24 (44.5)	24 (42.1)	1.171^b^	0.557
0.5–1.5	17 (28.3)	8 (14.8)	14 (24.6)		
≥1.5	24 (40.0)	22 (40.7)	19 (33.3)		
**Hb**^d^ **before chemo (g/L), no. (%)**					
< 60	2 (3.3)	5 (9.2)	2 (3.5)	0.206^b^	0.902
60–90	28 (46.7)	19 (35.2)	27 (47.4)		
≥90	30 (50.0)	30 (55.6)	28 (49.1)		
**Hb on the 7th day of chemo (g/L), no. (%)**					
< 60	14 (23.3)	6 (11.1)	3 (5.3)	8.649^b^	0.013
60–90	29 (48.3)	20 (37.0)	33 (57.9)		
≥90	17 (28.3)	28 (51.9)	21 (36.8)		
**Hb on the 14th day of chemo (g/L), no. (%)**					
< 60	3 (5.0)	3 (5.6)	5 (8.7)	3.072^b^	0.215
60–90	42 (70.0)	29 (53.7)	36 (63.2)		
≥90	15 (25.0)	22 (40.7)	16 (28.1)		
**Hb on the 21st day of chemo (g/L), no. (%)**					
< 60	1 (1.7)	1 (1.9)	5 (8.9)	1.759^b^	0.415
60–90	44 (73.3)	35 (64.8)	37 (66.1)		
≥90	15 (25.0)	18 (33.3)	15 (25.0)		
CRP^e^	30.7 [13.9, 79.8]	17.4 [12.6, 38.6]	29.6 [13.9, 63.2]	1.834^b^	0.133
PCT^f^	0.23 [0.11, 0.80]	0.21[0.11, 0.41]	0.15 [0.11, 0.40]	2.757^b^	0.399
IL-6^g^	51.3 [33.0, 70.9]	50.6 [32.0, 97.0]	55.7 [31.7, 106]	0.323^b^	0.891

^a^χ^2^, ^b^*H-*value; ^c^white blood cell; ^d^hemoglobin;^e^C-reactive protein; ^f^procalcitonin; ^g^Intedeukin-6.

### Primary outcome indicator

The main outcome measure was the incidence of perianal infection in patients with hematological malignancies, and binary logistic regression was used to adjust for diarrhea and its duration. There were statistically significant differences in the incidence of perianal infection among the control, the low-concentration intervention, and high-concentration groups (*P* = 0.004, *P* = 0.005, respectively). The probability of perianal infection in the low-concentration group was lower than that in the control group (ARR = 0.221, 95% CI 0.038–0.540). The OR for perianal infection was 0.144. In terms of absolute risk, the intervention led to an ARR of 22.1% (i.e., from 26.7% in controls to 5.6% in the intervention group). The probability of perianal infection in the high-concentration group was also lower than that in the control group (26.7% in the control group and 7.0% in the high-concentration group (ARR = 0.197, 95% CI 0.053–0.590). The risk of perianal infection in the high-concentration group was 19.7% lower compared to that in the control group (Table 4). The incidence of perianal infection in the low-concentration group was similar to that in the high-concentration group (*P* > 0.05).

**Table 4:** Main outcome indicator binary logistic regression.

**Group**	**No Perianal infection, *n* (%)**	**Occurrence of perianal infection, *n* (%)**	**OR (95% CI)**	**ARR (95% CI)**	***P-*value**
Control group (*n* = 60)	44 (73.3)	16 (26.7)	1	1	–
Low concentration group (*n* = 54)	51 (94.4)	3 (5.6)	0.144 (0.038–0.540)	0.221(0.038–0.540)	0.004
High concentration group (*n* = 57)	53 (93.0)	4 (7.0)	0.178 (0.053–0.590)	0.197 (0.053–0.590)	0.005

### Secondary outcome indicators

#### Severity of perianal infection

The distribution of perianal infection severity varied significantly across the groups (*P* < 0.001). In the control group (*n* = 60), 44 patients had no infection, 11 had Grade I, and 5 had Grade II or above. The low-concentration group (*n* = 54) had 51, 2, and 1 patient(s) in these respective categories, while the high-concentration group (*n* = 57) had 53, 3, and 1. The two intervention groups showed a comparable severity profile (*P* = 0.916). While the incidence of Grade II infections was not statistically different in pairwise comparisons between either intervention group and the control (*P* = 0.103 and *P* = 0.079, respectively), the aggregate proportion of these more severe infections was markedly lower in the intervention groups (low: 1.9%; high: 1.7%) than in the control group (8.3%).

#### Adverse events of perianal sitz bath

**Figure 2:**
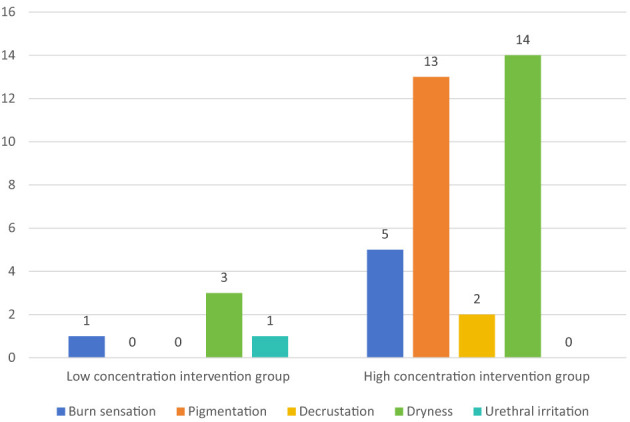
Comparison of specific adverse reactions between the low concentration intervention group and the high concentration intervention group.

Regarding perianal adverse events, none were observed in the control group. In the low-concentration intervention group, 5 events were reported by 4 patients, while in the high-concentration group, 34 events occurred among 20 patients. Importantly, no adverse events related to the sitz bath procedure itself were reported. Specifically, among the 4 patients in the low-concentration group, the adverse events comprised three cases of dry skin, one of perianal burning sensation, and one of urethral irritation. In the high-concentration group, the 34 events documented in 20 patients included: dry skin (*n* = 14), perianal hyperpigmentation (*n* = 13), perianal burning sensation (*n* = 5), and local skin desquamation (peeling; *n* = 2; Figure 2). After controlling for confounding factors, there was a statistically significant difference in the occurrence of adverse reactions between the two intervention groups (*P* < 0.05); the occurrence of adverse reactions in the high-concentration group was 4.7 times higher (95% CI 2.393–31.363, *p* = 0.001) than that in the low-concentration group (Supplementary 1).

## Discussion

### Baseline characteristic analysis

A total of 171 patients (114 women and 57 men) were included. The proportion of women in all three groups was >60%. The incidence of male patients with hematological malignancies is higher than that of female patients, and the proportion of male patients with perianal disease is higher than that of female patients (13, 14). According to a comprehensive evaluation, men were better able to meet the inclusion criteria of this study, but more women were included in this study. This may be because more women than men were willing to participate. Moreover, owing to the impact of daily living habits, physiological periods, and other factors, female patients are more willing to clean and observe private areas such as the perianus and vulva. Among the patients, 158 were ≤ 60 years old, which may be because middle-aged and young people in China show a high incidence of anorectal diseases (15), and being ≤ 60 years old is also a risk factor for infection. In addition, the main caregivers of older patients were their children, who were influenced by traditional concepts and did not expect their children to assist them in the care of their private parts. In addition, older patients' self-care ability decreased because of bleeding and fatigue symptoms caused by bone marrow suppression after chemotherapy, which also affected their participation in this study. In addition, a statistically significant difference in educational levels was observed among the three patient groups. Educational attainment can influence patient compliance through several pathways: it often correlates with health literacy, affecting the comprehension of medical instructions and it can impact the ability to accurately recognize and report early symptoms. These factors collectively could theoretically affect daily hygiene practices and, consequently, the risk and severity of perianal infection. To mitigate such variability, this study implemented a rigorously unified and standardized perianal care protocol for all participants. The protocol was designed to minimize dependence on baseline knowledge by providing clear, step-by-step visual and verbal instructions, along with direct supervision during initial training and periodic checks. This structured approach likely ensured a high and consistent level of adherence across all educational backgrounds within the trial setting. Consequently, under this controlled nursing framework, educational level did not emerge as a significant factor affecting the incidence of perianal infection in this study.

### Disease-related characteristics

Lymphoma accounts for the highest proportion of hematological malignancies, and the number of new cases each year is higher than that of leukemia (16, 17), but more than 80% of the patients were diagnosed with acute leukemia. The possible reasons for this discrepancy were that the differences between leukemia and lymphoma chemotherapy, including the duration of chemotherapy and the use of drug doses, were inconsistent. Patients with lymphoma were most likely to complete chemotherapy in 1 day, whereas those on leukemia chemotherapy had longer therapy durations. In addition, chemotherapy regimens for leukemia used higher doses of drugs, such as the DA regimens commonly used for leukemia and CHOP regimens commonly used for lymphoma. Leukemia chemotherapy usually involves drugs with stronger bone marrow inhibition (such as cytarabine) (18, 19). Simultaneously, with the improvement of medical technology and treatment methods, many patients with lymphoma could be treated at home, because the duration of myelosuppression and hospitalization was shorter, and the incidence of infection in hospitalized patients with lymphoma receiving chemotherapy was reduced, resulting in a small sample size. In addition, patients with complex and difficult-to-treat conditions might choose the new CAR-T therapy after remission of consolidation chemotherapy (20, 21); therefore, the proportion of patients who continued chemotherapy in the ward would be reduced. This study required patients to take a 14-day sitz bath, which patients with severe dependence on self-care ability find difficult to complete; therefore, those with moderate-to-severe dependence comprised only 4.7% of the population. In addition, the incidence of diarrhea and constipation was 25.1% and 36.3%, respectively, which was similar to that in previous results (22, 23) and might be related to the use of drugs, changes in diet or lifestyle, and bone marrow suppression after chemotherapy. Among the patients with diarrhea, the incidence of diarrhea in the control group (36.7%) was significantly higher than that in the low- (22.2%) and high-concentration groups (15.8%), which might be led to persistent moisture around the anus, which provides an environment for bacterial growth, and prolonged diarrhea causes perianal skin damage, both of which increase the likelihood of perianal infection. Therefore, both diarrhea and duration of diarrhea were controlled in the analysis of outcome indicators in this study. Among the included patients, the proportion of patients with a history of hemorrhoids, anal fissure, and perianal infection was consistent with the results of an epidemiological investigation of anorectal diseases (15), which might be because the population included in this study had high-risk factors for perianal infection, and a history of hemorrhoids and perianal infection have been confirmed to be influencing factors for perianal infection.

### Analysis of laboratory test results

The laboratory results of the included patients were collected before and after chemotherapy. Before chemotherapy, there were no differences in the albumin level, original proportion of blood cells, white blood cell count, neutrophil count, and CRP level among the three groups, indicating that there were no differences in the nutritional level, tumor burden, infection status, or bone marrow suppression level among the three groups before chemotherapy. There were statistically significant differences in white blood cell, neutrophil, and hemoglobin counts within 1 week after chemotherapy among the three groups, which might be caused by the different sensitivities and drug resistance of individuals to chemotherapy drugs. The white blood cell, neutrophil, and hemoglobin counts and the three inflammatory indicator (CRP, PCT, and IL-6) levels did not differ at 14 and 21 days after chemotherapy. The duration of myelosuppression, time of bone marrow recovery, or infection status among the three groups after chemotherapy did not differ. Moreover, the changing trend in routine blood examination results in this study indicated that patients with hematologic malignancies entered the myelosuppression period 7–14 days after chemotherapy and that the bone marrow gradually recovered after reaching the lowest point (16).

### Incidence of perianal infection

The incidence of perianal infection in the low- and high-concentration groups was similar, but lower than that in the control group, suggesting that PI-containing Sitz baths could effectively prevent perianal infection during chemotherapy in patients with hematologic malignancies.

The reasons might be as follows: (1) all patients involved in the study were given targeted health guidance, such as a reasonable diet to reduce the occurrence of constipation and diarrhea to reduce the risk factors for perianal infection; and (2) perianal cleaning and warm water was used to promote perianal local blood circulation and promote the healing of inflammation (24) before the sitz bath. Additionally, warm water can help in defecation and reduce the incidence of anal fissures. (3) Diluted PI can disinfect the perianal area and kill microorganisms; it has anti-inflammatory, antiseptic, analgesic, and antipruritic properties, promotes granulation tissue growth (25), and ensures that patients maintain perianal asepsis during a period of severe bone marrow suppression, thus effectively reducing the occurrence of perianal infection (26). (4) There was no difference in the incidence of perianal infection between the high-concentration and low-concentration groups, which might be because a low concentration can achieve effective disinfection and sterilization of the perianal area, consistent with the findings of Lu et al. (10).

### Comparison of the degree of perianal infection

Although the severity of perianal infection in the intervention group was lower than that in the control group, the severities of perianal infection did not differ between the low- and high-concentration groups.

This could be because: (1) warm water could facilitate blood circulation and promotehealing of the inflammation to avoid further serious infection (27); (2) the PI disinfection effect reduced the severity of infection (10); (3) strict perianal observation could facilitate timely intervention for the symptoms of infection to avoid the deterioration of infection; and, (4) there was no difference in the incidence of grade II and above infections among the three groups, which may be due to the small sample size.

### Analysis of perianal adverse reaction

The high-concentration group was more likely to experience adverse reactions, the most common being local skin dryness and perianal pigmentation.

The reasons might include the following: (1) Patients with severe anemia or moderate-to-severe self-care ability need to be accompanied by family members for sitz baths (28). The height of the sitz bath was kept at 40 cm, consistent with the height of the ward seat; the temperature of the sitz bath was maintained at 40–45 °C; and the sitz bath duration was fixed at 10 min to reduce or avoid the occurrence of adverse events such as burns and falls during the sitz bath. (2) The high-concentration sitz bath had stronger disinfection ability, which would weaken the skin and increase water loss; therefore, the occurrence of peeling and skin dryness would increase in the higher-concentration intervention group. Patients avoid the anal area and use moisturizer to relieve symptoms, and patients with peeling should be moved to the low-concentration group in time according to the symptoms and recommended to use moisturizers to heal the peeling site. (3) The PI solution was yellowish brown, and a long-term sitz bath stained the skin of patients; therefore, the hyperpigmentation in the high-concentration group was significantly higher than that in the low-concentration group. However, 1–2 months after the sitz bath, the patient's skin returned to its pre-intervention state.

### Limitations

Several limitations should be considered when interpreting the results of this study. First, two initially planned outcome measures—anal swab culture and pain assessment—were omitted following an interim analysis. Their exclusion means we cannot identify causative pathogens or evaluate effects on patient-reported quality of life, thereby limiting conclusions to clinically observable signs and complications. This issue may partly stem from the single-center design and modest sample size, which could also reduce the ability to detect other outcomes, such as additional adverse events. Future multi-center studies with larger samples are needed to clarify the relevance of these omitted indicators. Second, the relatively small sample size may have limited the statistical power to detect potential differences in the incidence and severity of perianal infection between the two intervention groups, which appeared numerically similar in our analysis. Third, the diagnosis of perianal infection, the primary endpoint, relied primarily on clinical assessment rather than objective imaging modalities such as ultrasound or MRI. The use of imaging could have provided more accurate and reproducible diagnostic confirmation.

## Conclusions

Sitz baths with concentrations of 1:50 and 1:200 diluted PI can effectively reduce the incidence and severity of perianal infection in patients with hematological malignancies during chemotherapy compared to routine perianal cleaning care. However, there was no difference in the effectiveness of sitz baths with concentrations of 1:50- and 1:200-diluted PI in preventing perianal infection between the two groups, which requires a large sample size to confirm. In addition, the occurrence of adverse perianal reactions in the 1:200-diluted PI sitz bath was lower than that in the 1:50 diluted PI sitz bath.

## Data Availability

The raw data supporting the conclusions of this article will be made available by the authors, without undue reservation.
