# Supplementary material for: Sitz bath with different concentrations of diluted povidone-iodine for prevention of perianal infection in patients with hematological malignancies undergoing chemotherapy: a randomized controlled trial in a tertiary hospital in China
Source: Front Public Health. 2026 Jan 29;14:1743662. doi: 10.3389/fpubh.2026.1743662 (PMC12894220; doi:10.3389/fpubh.2026.1743662)
Supplement: Supplementary file 1 [file Supplementary_file_1.docx]

**Supplementary file 1.Comparison of adverse reactions between the low-concentration intervention group and the high-concentration intervention group**

| Group | No AEs, n(%) | AEs, n(%） | *OR*  (95% CI) | *RR*  (95% CI) | *X^2^* | *P*  Value |
| --- | --- | --- | --- | --- | --- | --- |
| Low concentration group（n=54） | 50 (92.6) | 4 (7.41) | 8.663  (2.393-31.363) | 4.737  (2.393-31.363) | 10.957 | .001 |
| High concentration group（n=57） | 37 (64.9) | 20 (35.1) |  |  |  |  |
