# Supplementary material for: Sitz bath with different concentrations of diluted povidone-iodine for prevention of perianal infection in patients with hematological malignancies undergoing chemotherapy: a randomized controlled trial in a tertiary hospital in China
Source: Front Public Health. 2026 Jan 29;14:1743662. doi: 10.3389/fpubh.2026.1743662 (PMC12894220; doi:10.3389/fpubh.2026.1743662)
Supplement: Supplementary file 2 [file Supplementary_file_2.docx]

**Table 1.Perianal sitz bath inspection list (for patients)**

| Time | Water temperature | Whether perianal cleansing or sitz bath is completed  (√ or ×) |
| --- | --- | --- |
|  |  |  |
|  |  |  |

**Table 2.Perianal checklist (for researchers)**

Bed Number: Admission Number (AD):

| Date | Perianal condition | Inspector (signed by two) |
| --- | --- | --- |
|  |  |  |
|  |  |  |

*Judging the degree of perianal infection: Grade Ⅰ : redness, swelling, heat and pain limitation; Grade Ⅱ : obvious redness, swelling, heat and pain, abscess formation, the top of the lesion felt fluctuation; Grade III: skin ulceration, necrosis with infection, bleeding, sinus or wound formation; If no infection occurs draw“-”.

* Perianal adverse reactions: (if any, write the code in the table)

Such as: A, perianal skin burning sensation; B, pigmentation; C, perianal skin peeling; D, dry skin;E, irritation of urethral meatus; F, other (please explain)
