# Supplementary material for: Sitz bath with different concentrations of diluted povidone-iodine for prevention of perianal infection in patients with hematological malignancies undergoing chemotherapy: a randomized controlled trial in a tertiary hospital in China
Source: Front Public Health. 2026 Jan 29;14:1743662. doi: 10.3389/fpubh.2026.1743662 (PMC12894220; doi:10.3389/fpubh.2026.1743662)
Supplement: Supplementary file 3 [file Table_3.doc]

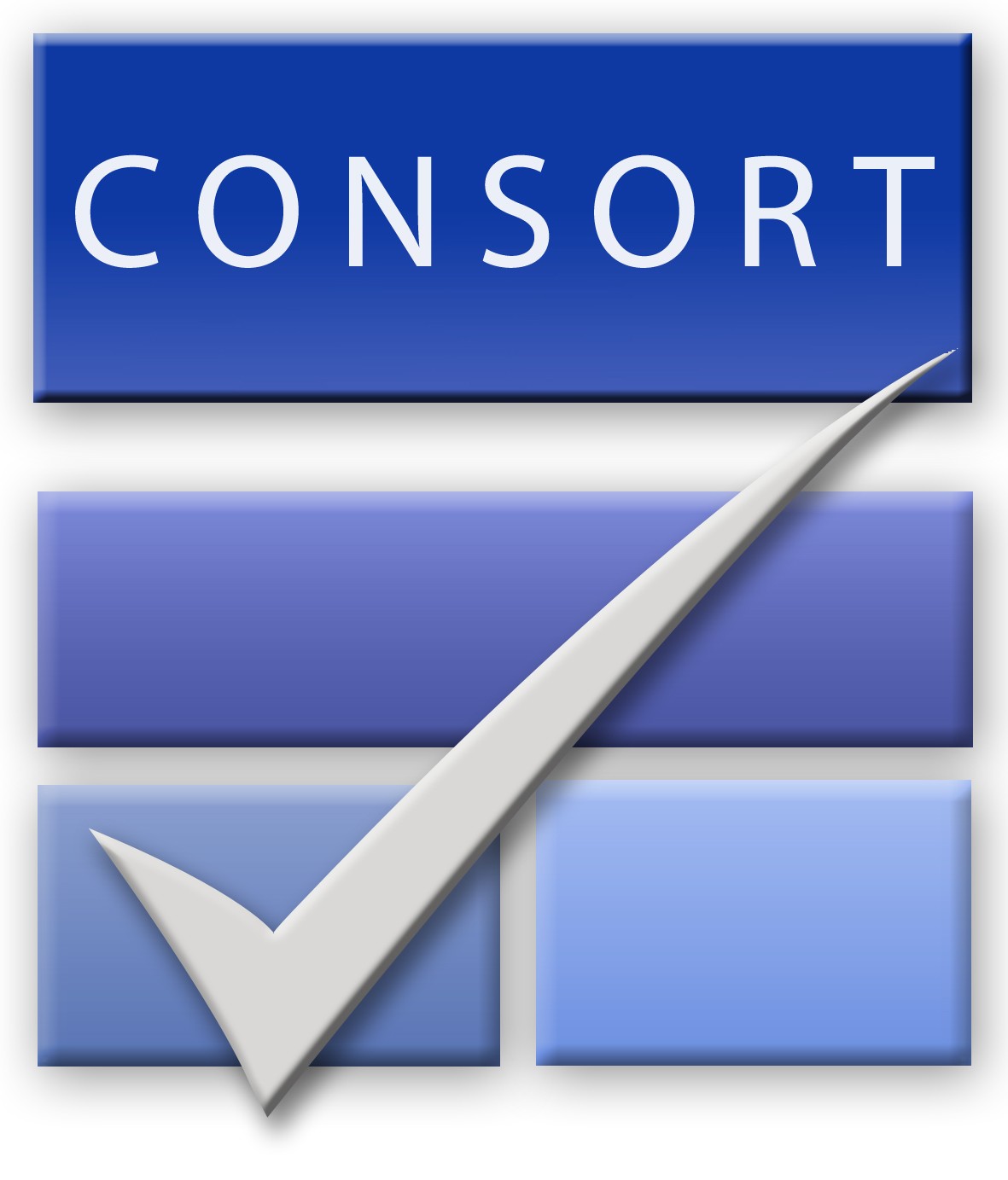
CONSORT 2010 checklist of information to include when reporting a randomised trial*

| Section/Topic | Item No | Checklist item | Reported on page No |
| --- | --- | --- | --- |
| Title and abstract | | | |
|  | 1a | Identification as a randomised trial in the title | Page 1 line 4-20 |
|  | 1b | Structured summary of trial design, methods, results, and conclusions (for specific guidance see CONSORT for abstracts) | Page 1 line 32-58 |
| Introduction | | | |
| Background and objectives | 2a | Scientific background and explanation of rationale | Page 2 line 137-139，193-211 |
|  | 2b | Specific objectives or hypotheses | Page 2 line 206-211 |
| Methods | | | |
| Trial design | 3a | Description of trial design (such as parallel, factorial) including allocation ratio | Page 2 line 217-222 |
|  | 3b | Important changes to methods after trial commencement (such as eligibility criteria), with reasons | Page 3 line 295-317 |
| Participants | 4a | Eligibility criteria for participants | Page 2-3 line 228-236 |
|  | 4b | Settings and locations where the data were collected | Page 2 line 220-222 |
| Interventions | 5 | The interventions for each group with sufficient details to allow replication, including how and when they were actually administered | Page 4 line 385-413 |
| Outcomes | 6a | Completely defined pre-specified primary and secondary outcome measures, including how and when they were assessed | Page 4 line 421-464 |
|  | 6b | Any changes to trial outcomes after the trial commenced, with reasons | Page 3 line 308-313 |
| Sample size | 7a | How sample size was determined | Page 3 line 262-282 |
|  | 7b | When applicable, explanation of any interim analyses and stopping guidelines | n/a, not involved |
| Randomisation: |  |  |  |
| Sequence generation | 8a | Method used to generate the random allocation sequence | Page 3 line 322-342 |
|  | 8b | Type of randomisation; details of any restriction (such as blocking and block size) | Page 3 line 322-342 |
| Allocation concealment mechanism | 9 | Mechanism used to implement the random allocation sequence (such as sequentially numbered containers), describing any steps taken to conceal the sequence until interventions were assigned | Page 3-4 line 322-353 |
| Implementation | 10 | Who generated the random allocation sequence, who enrolled participants, and who assigned participants to interventions | Page 3-4 line 322-353 |
| Blinding | 11a | If done, who was blinded after assignment to interventions (for example, participants, care providers, those assessing outcomes) and how | Page 3-4 line 322-353 |
|  | 11b | If relevant, description of the similarity of interventions | n/a, not involved |
| Statistical methods | 12a | Statistical methods used to compare groups for primary and secondary outcomes | Page 5 line 467-525 |
|  | 12b | Methods for additional analyses, such as subgroup analyses and adjusted analyses | n/a, not involved |
| Results | | | |
| Participant flow (a diagram is strongly recommended) | 13a | For each group, the numbers of participants who were randomly assigned, received intended treatment, and were analysed for the primary outcome | Page 4 line 385-413 |
|  | 13b | For each group, losses and exclusions after randomisation, together with reasons | Figure 1 |
| Recruitment | 14a | Dates defining the periods of recruitment and follow-up | Page 2 line 221-211 |
|  | 14b | Why the trial ended or was stopped | Page 3 line 281-282 |
| Baseline data | 15 | A table showing baseline demographic and clinical characteristics for each group | Table 1 |
| Numbers analysed | 16 | For each group, number of participants (denominator) included in each analysis and whether the analysis was by original assigned groups | page 5 line 467-525 |
| Outcomes and estimation | 17a | For each primary and secondary outcome, results for each group, and the estimated effect size and its precision (such as 95% confidence interval) | Page 9-10 line 968-1090 |
|  | 17b | For binary outcomes, presentation of both absolute and relative effect sizes is recommended | Table 2-4 |
| Ancillary analyses | 18 | Results of any other analyses performed, including subgroup analyses and adjusted analyses, distinguishing pre-specified from exploratory | n/a, not involved |
| Harms | 19 | All important harms or unintended effects in each group (for specific guidance see CONSORT for harms) | n/a, not involved |
| Discussion | | | |
| Limitations | 20 | Trial limitations, addressing sources of potential bias, imprecision, and, if relevant, multiplicity of analyses | Page 12 line 1095-1314 |
| Generalisability | 21 | Generalisability (external validity, applicability) of the trial findings | Page 12 line 1301-1305 |
| Interpretation | 22 | Interpretation consistent with results, balancing benefits and harms, and considering other relevant evidence | Page 10-12 line 1093-1290 |
| Other information | | |  |
| Registration | 23 | Registration number and name of trial registry | Page 3 line 292-293 |
| Protocol | 24 | Where the full trial protocol can be accessed, if available | Page 3 line 284-286 |
| Funding | 25 | Sources of funding and other support (such as supply of drugs), role of funders | Page 12 line 1359-1367 |

*We strongly recommend reading this statement in conjunction with the CONSORT 2010 Explanation and Elaboration for important clarifications on all the items. If relevant, we also recommend reading CONSORT extensions for cluster randomised trials, non-inferiority and equivalence trials, non-pharmacological treatments, herbal interventions, and pragmatic trials. Additional extensions are forthcoming: for those and for up to date references relevant to this checklist, see [www.consort-statement.org](http://www.consort-statement.org/).
